# Effectiveness and Safety of DOACs vs. Warfarin in Patients With Atrial Fibrillation and Frailty: A Systematic Review and Meta-Analysis

**DOI:** 10.3389/fcvm.2022.907197

**Published:** 2022-06-24

**Authors:** Shan Zeng, Yuxiang Zheng, Jingzhou Jiang, Jianyong Ma, Wengen Zhu, Xingming Cai

**Affiliations:** ^1^Key Laboratory of Prevention and Treatment of Cardiovascular and Cerebrovascular Diseases, Department of Cardiology, First Affiliated Hospital, Ministry of Education, Gannan Medical University, Ganzhou, China; ^2^Second Clinical Medical College, Nanchang University, Nanchang, China; ^3^Department of Cardiology, The First Affiliated Hospital of Sun Yat-sen University, Guangzhou, China; ^4^Department of Pharmacology and Systems Physiology, University of Cincinnati College of Medicine, Cincinnati, OH, United States; ^5^Department of Geriatric, The First Affiliated Hospital of Sun Yat-sen University, Guangzhou, China

**Keywords:** atrial fibrillation, frailty, anticoagulation, prognosis, meta-analysis

## Abstract

**Background:**

Patients with atrial fibrillation (AF) and frailty are a considerable group in clinical practice. However, existing studies provide insufficient evidence of anticoagulation strategies for these patients. Therefore, we conducted a meta-analysis to determine the effectiveness and safety outcomes of direct oral anticoagulants (DOACs) for these patients.

**Methods:**

Randomized controlled trials or observational studies reporting the data about the DOACs and warfarin therapy among frail AF patients were included. The search was performed in the PubMed and Embase databases up to March 2022. Frailty was defined using the most widely used claims-based frailty index or the cumulative deficit model-based frailty index.

**Results:**

A total of 4 studies involving 835,520 patients were included. Compared with warfarin, DOACs therapy reduced the risks of stroke or systemic embolism (HR = 0.79, 95%CI: 0.69–0.90), ischemic stroke (HR = 0.79, 95%CI: 0.71–0.87), hemorrhagic stroke (HR = 0.52, 95%CI: 0.35–0.76), and all-cause death (HR = 0.90, 95%CI: 0.84–0.96). In safety outcomes, DOACs was significantly associated with reduced risks of major bleeding (HR = 0.79, 95%CI: 0.64–0.97) and intracranial hemorrhage (HR = 0.58, 95%CI: 0.52–0.65) compared to warfarin, but there were no statistically differences in gastrointestinal bleeding (HR = 0.97, 95%CI: 0.73–1.29).

**Conclusions:**

DOACs exerted superior effectiveness and safety outcome than warfarin in AF patients with frailty.

## Introduction

Atrial fibrillation (AF) is the most common arrhythmia among adults affecting millions of people worldwide (1). Due to the disorganization of atrial contraction, the blood flow in AF patients is pooling and stasis, leading to a significant increase in the risk of thromboembolic events (2). Frailty is a multisystem clinical syndrome characterized by decreased physiological reserve and diminished stress capacity. The deterioration of multiple physiological systems complicates medical treatment and rehabilitation, leading to poor health outcomes (3–5). Recent investigations have demonstrated that the prevalence of AF and frailty increases with age and often occurs simultaneously (6). AF patients with frailty face difficulties in clinical treatment because of their multiple comorbidities and medications.

Although frailty is associated with increased stroke and mortality in patients with AF, there is evidence of a risk-treatment paradox, whereby frail patients with a higher risk of complications from AF are less likely to use oral anticoagulants (OACs) than non-frail patients (7, 8). Existing studies confirm that most frail AF patients should receive OACs to reduce stroke or systemic embolism (SSE) risk because the benefit outweighs the risk of bleeding (9). Clinical use of specific OACs may be based on age and/or comorbidity patterns (often in association with weight, renal function, and history of bleeding) (10). However, chronological age is an outdated concept compared to biological or functional age. In this new definition, frailty plays an indispensable role that cannot be ignored and is increasingly being used to guide the care of older adults (11). As the first-line choice for non-valvular AF patients, direct oral anticoagulants (DOACs) consist of direct thrombin (FIIa) inhibitors or direct factor Xa (FXa) inhibitors, including dabigatran, apixaban, rivaroxaban, edoxaban. Previous studies have shown that DOACs are safe and effective in patients with AF (12–16), including old patients with non-valvular atrial fibrillation (NVAF) (17). Compared with warfarin, the advantages of DOACs included a wider therapeutic window, rapid onset of action, stable and predictable anticoagulation effects, and limited drug interactions. So, it may be a better anticoagulant choice for frailty patients. Due to the poor representation of frail adults and the lack of frailty assessment in clinical trials (18), there is still no consensus on the choice of anticoagulants in frail AF patients. It is uncertain whether DOACs have an advantage over warfarin.

To fill this gap, we conducted a systematic review and meta-analysis to better understand the effectiveness and safety of DOACs in AF patients with frailty, as an increasing number of updated studies have been published.

## Methods

We conducted this meta-analysis based on the criteria of the Cochrane Handbook for Systematic Reviews of Interventions (version 6.2). The results were presented according to the preferred reporting items for systematic review and meta-analysis (PRISMA) 2020 statement. Ethical approval was not required, as this study only included articles of published data in the public domain.

### Literature Search

Two reviewers performed the literature search, systematically searching the PubMed and Embase database sources until March 2022 for studies exploring the effectiveness and safety of DOACs compared with Warfarin in AF patients with frailty. The following search terms were used: (1) “atrial fibrillation,” (2) “dabigatran” OR “rivaroxaban” OR “apixaban” OR “edoxaban” OR “non-vitamin K antagonist oral anticoagulant” OR “direct oral anticoagulant” OR “novel oral anticoagulant” OR “NOAC” OR “DOAC”, (3) “frail” OR “frailty” OR “frailness” OR “Frailty Syndrome,” (4) “Vitamin K antagonists” OR “VKA” OR “warfarin” OR “dicoumarol” OR “acenocoumarol” OR “Coumadin,” The above four categories of search terms were combined using the Boolean operator “and.” The detailed search strategies are shown in Supplementary Table 1. In addition, the reference lists of the retrieved articles and prior reviews were manually checked for additional eligible studies. We applied no linguistic restrictions in the literature search.

### Inclusion and Exclusion Criteria

Criteria for inclusion were as follows: (1) The study was a randomized controlled trial (RCT), *post-hoc* analyses of RCT or observational (prospective or retrospective cohort) study; (2) The study included AF patients with frailty who received warfarin or DOACs (dabigatran, rivaroxaban, apixaban, or edoxaban); (3) Quantitative estimates of the hazard ratios (HRs) and 95% confidence intervals (CIs) reporting for safety and effectiveness outcomes among patients. Additionally, articles using claims-based frailty index or cumulative deficit model-based frailty index were included. Where frailty status was dichotomized, the threshold used by the study author was used.

We excluded studies focusing on AF patients without a clear systemic definition of frailty. Studies without adjustment or with a sample size of < 100 were excluded. In addition, we also excluded certain publication types (e.g., reviews, comments, case reports, case series, letters, editorials, and meeting abstracts) due to insufficient data or study details. If there were overlapping data among two or more studies, we included the one with the largest sample size or the longest follow-up duration.

### Study Selection and Data Abstraction

Two reviewers independently screened the titles and abstracts of the retrieved studies from the electronic databases. Subsequently, we selected the eligible studies after the full-text screenings based on the pre-defined inclusion criteria. Disagreements were resolved by discussion between two reviewers or consultation with the corresponding authors. The following data of the included studies were abstracted: study characteristics (first author, year of publication, study design), study population, and baseline characteristics (age, male ratio, sample size, stroke and bleeding risk prediction scores, drugs in the DOACs group, definition of frailty, history of stroke and bleeding), effectiveness and safety outcomes, follow-up period, and outcome data (sample size and the number of events between groups, adjusted HRs).

### Study Quality Assessment

We assessed the quality of *post-hoc* analysis of RCTs or observational cohorts by using the Newcastle-Ottawa Scale (NOS) tool. This tool had three domains with a total of nine points: the selection of cohorts (0–4 points), the comparability of cohorts (0–2 points), and the assessment of the outcome (0–3 points). In this meta-analysis, the NOS of ≥6 and <6 points were moderate-to-high quality and low-quality, respectively (19).

### Statistical Analysis

This meta-analysis's statistical analyses were conducted using the Review Manager version 5.4 software (the Cochrane Collaboration 2014, Nordic Cochrane Center Copenhagen, Denmark; https://community.cochrane.org/). In this study, significant heterogeneity was indicated by a *P*-value of <0.10 in the Cochrane Q test or an *I*^2^ value of > 50%, which led to the use of random-effects models and the exploration of a potential source of heterogeneity. When these tests were negative for heterogeneity, fixed-effects models were chosen to calculate pooled HRs through the inverse-variance method. In the pooled analysis, the adjusted HRs and 95% CIs were converted to the natural logarithms [Ln [HR]] and their corresponding standard errors [Ln [upper CI]-Ln [lower CI]/3.92], which were pooled by a DerSimonian and Laird random-effects model with an inverse variance method.

## Results

### Study Selection

**Figure 1:**
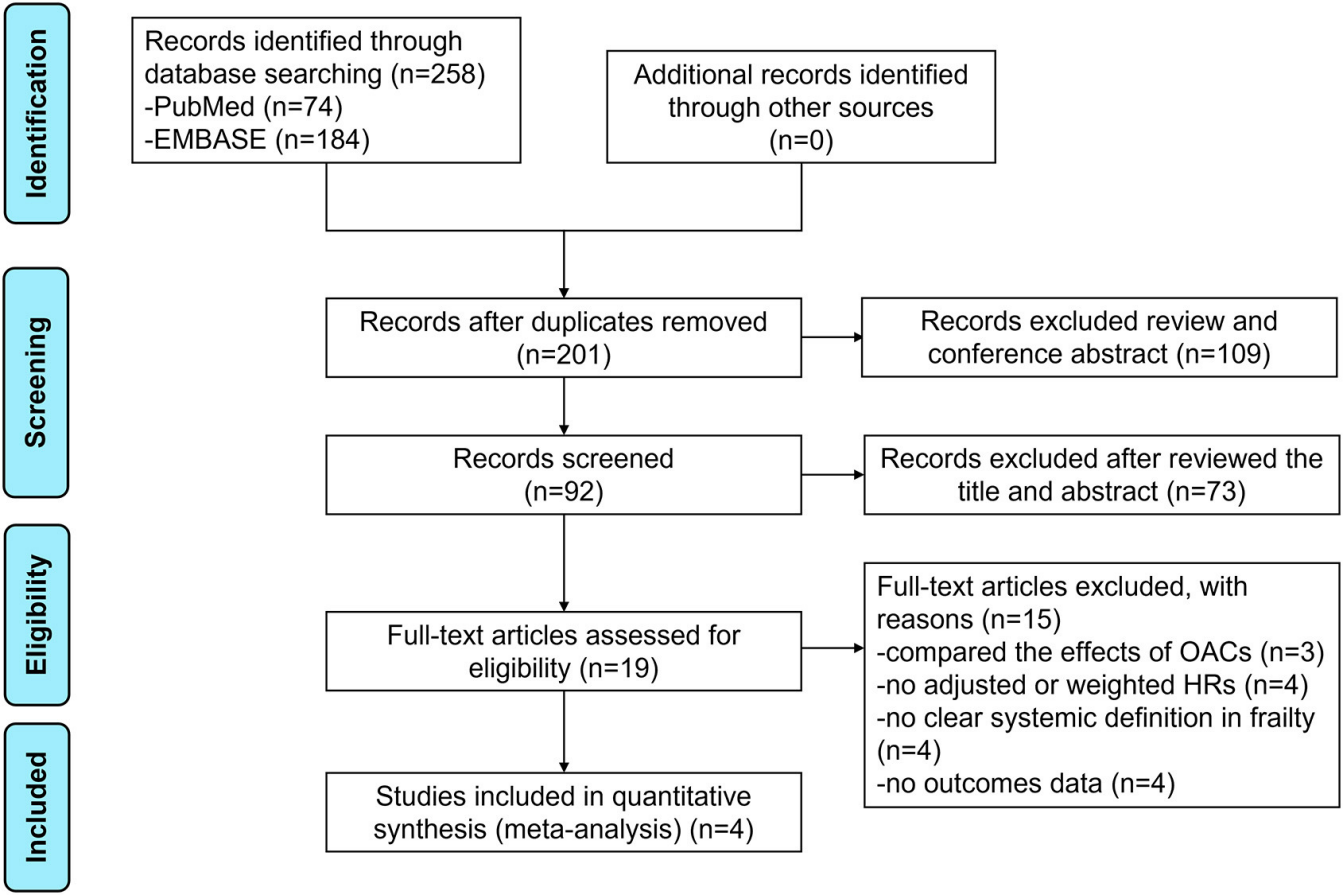
The flow chart of literature retrieval of this meta-analysis.

The flow chart of literature retrieval is presented in Figure 1. Through searching the electronic searches in the PubMed and EMBASE databases, our initial search yielded 258 articles. After the records screening, we selected 92 relevant articles. By reviewing the abstract, 19 remaining studies were potentially available, and further assessed under the full-text screenings. According to the pre-defined inclusion and exclusion criteria, we subsequently excluded 15 studies because (1) studies compared the effects of OACs (*n* = 3); (2) studies did not report adjusted or weighted HRs (*n* = 4); (3) studies did not report a clear systemic definition in frailty (*n* = 4); (4) studies did not report the studied outcomes (*n* = 4). Finally, a total of 4 studies (1 *post-hoc* analyses of RCTs and 3 observational studies) were included in our meta-analysis (20–23).

### Baseline Characteristics

Baseline characteristics of the included studies are illustrated in Table 1. Among the included studies, 3 were from the United States of America, and 1 from multiple countries (America, Europe, Asia–Pacific region, and South Africa). The mean age of patients ranged from 77.3 to 86.0 years, and the sample size was from 10,754 to 653,421. Three of the included articles used a claims-based frailty index and one article used a cumulative deficit model-based frailty index. Across studies, the study populations in the DOACs group were administrated with dabigatran, apixaban, rivaroxaban, and edoxaban. Risk of bias evaluation was performed, shown in Supplementary Table 2. All the studies had a NOS of ≥6 points suggesting moderate-to-high quality.

**Table 1:** Baseline characteristics of the included studies in this meta-analysis.

	**Martinez et al**. **(****22****)**			**Kim et al**. **(****20****)**			**Lip et al**. **(****21****)**			**Wilkinson et al**. **(****23****)**	
**Group**	**API/**	**DA/**	**RIV/**	**API/**	**DA/**	**RIV/**	**API/**	**DA/**	**RIV/**	**EDO (30 mg)/**	**EDO (60 mg)/**
	**Warfarin**	**Warfarin**	**Warfarin**	**Warfarin**	**Warfarin**	**Warfarin**	**Warfarin**	**Warfarin**	**Warfarin**	**Warfarin**	**Warfarin**
Participants (N)	1,392/ 1,392	1,350/ 1,350	2,635/ 2,635	109,369/ 109,369	79,365/ 79,365	137,972/ 137,972	34,594/ 34,594	9,263/ 9,263	39,898/ 39,898	5,483/ 5,478	5,447/ 5,478
Study design	Observational Study			Observational Study			Observational Study			*Post-hoc* analysis of ENGAGE AF-TIMI48	
Region	America			America			America			Multi-center (America, Europe, Asia–Pacific region, and South Africa)	
Age (mean, y)	86.0/86.0 (median)	85.0/86.0 (median)	85.0/86.0 (median)	77.3/77.3	76.4/76.4	76.8/76.8	84.2/84.2	83.3/83.4	83.7/83.7	NA	NA
Male ratio (%)	63.7/62.8	64.7/62.7	65.2/64.4	49.6/49.4	50.1/50.1	50.1/50.1	35.0/35.2	35.3/35.5	35.6/35.5	60.5/60.7	60.5/60.7
HAS-BLED	2.0/2.0	2.0/2.0	2.0/2.0	2.1/2.1	2.0/2.0	2.1/2.1	3.7/3.7	3.6/3.6	3.7/3.6	NA	NA
CHA2DS2-VASc	4.0/4.0	4.0/4.0	4.0/4.0	4.2/4.2	4.1/4.1	4.1/4.1	5.1/5.1	5.1/5.1	5.1/5.1	NA	NA
Stroke history	18.2/18.0	15.2/16.8	15.0/15.7	5.5/5.6 (inpatient)	4.6/4.6 (inpatient)	4.7/4.8 (inpatient)	22.3/22.2	21.4/22.3	21.8/22.0	NA	NA
Bleeding history	3.4/3.1	1.3/1.4	2.7/2.4	2.5/2.6 (inpatient)	1.5/1.5 (inpatient)	2.0/2.0 (inpatient)	25.7/25.9	24.5/25.2	26.0/26.3	NA	NA
Follow-up	2 years			84 days	72 days	82 days	183 days/ 233 days	226 days/ 235 days	220 days/ 234 days	2.8 years	
Definition of frailty	CFI ≥ 0.20			CFI ≥ 0.15			CFI ≥ 0.20			FI ≥ 0.12	

*DA, dabigatran; RIV, rivaroxaban; API, apixaban; EDO, edoxaban; HAS-BLED, Hypertension, Abnormal Renal/Liver Function, Stroke, Bleeding History or Predisposition, Labile International Normalized Ratio, Elderly, Drugs/Alcohol; CHA2DS2-VASc, congestive heart failure, hypertension, age ≥75 y (doubled), diabetes mellitus, stroke (doubled)-vascular disease, age 65–74 and sex category (female); CFI, claims-based frailty index; FI, frailty index*.

## Synthesis of Results

### Outcomes Between DOACs vs. Warfarin in Frail AF Patients

**Figure 2:**
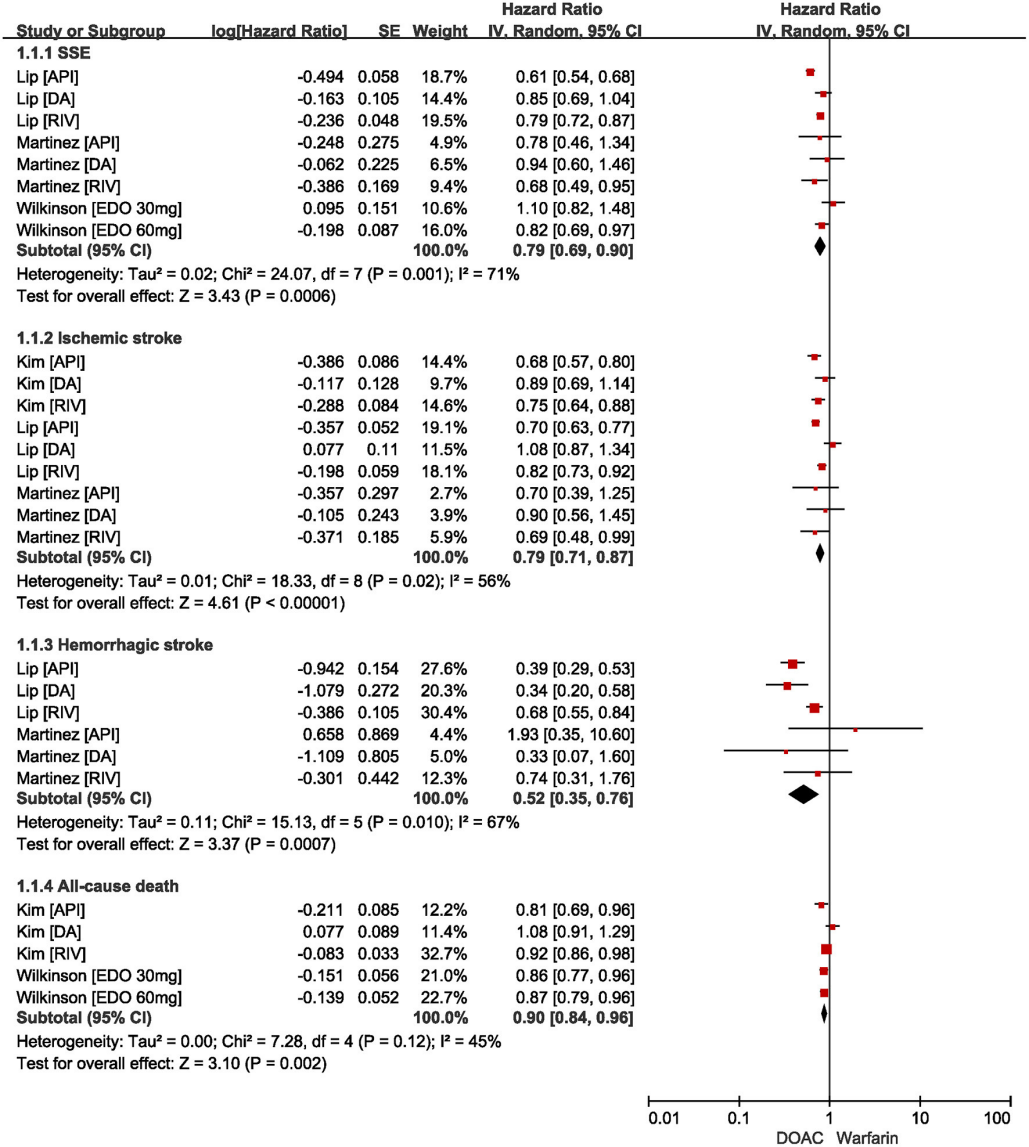
Comparing effectiveness of DOACs with warfarin in AF patients with frailty. AF, atrial fibrillation; SSE, stroke and systemic embolism; DOACs, direct oral anticoagulants; CI, confidence interval; IV, inverse of the variance; SE, standard error.

As shown in Figure 2, our pooled results based on the random-effects model showed that compared with warfarin, the use of DOACs was significantly associated with reduced risks of effectiveness outcomes, including SSE (HR = 0.79, 95%CI: 0.69–0.90), ischemic stroke (HR=0.79, 95%CI: 0.71–0.87), hemorrhagic stroke (HR = 0.52, 95%CI: 0.35–0.76), and all-cause death (HR = 0.90, 95%CI: 0.84–0.96).

**Figure 3:**
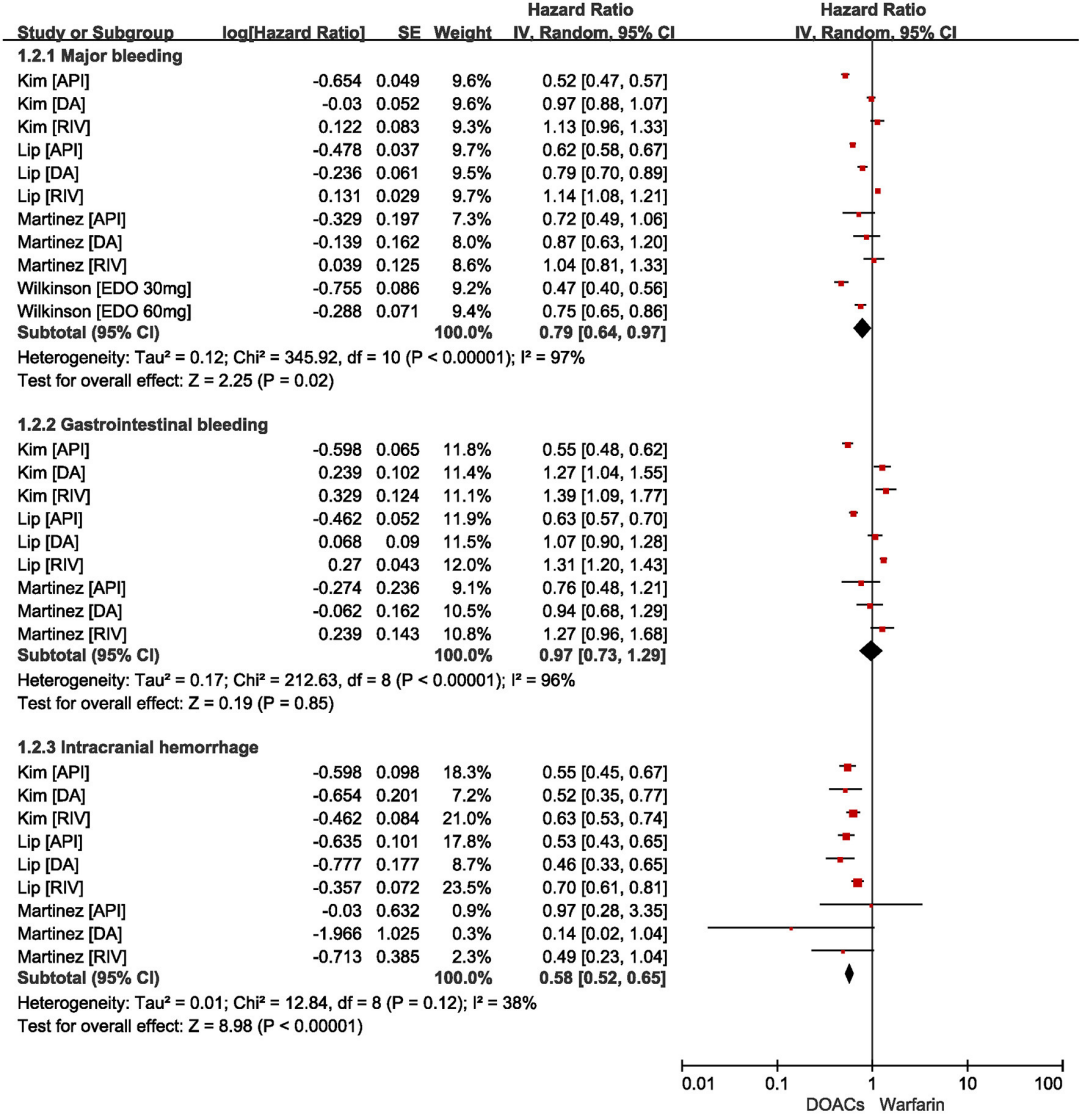
Comparing safety of DOACs with warfarin in AF patients with frailty. AF, atrial fibrillation; DOACs, direct oral anticoagulants; CI, confidence interval; IV, inverse of the variance; SE, standard error.

The safety outcomes are shown in Figure 3. Compared with warfarin users, DOACs were significantly associated with reduced risks of major bleeding (HR = 0.79, 95%CI: 0.64–0.97) and intracranial hemorrhage (HR = 0.58, 95%CI: 0.52–0.65). There were no statistical differences in gastrointestinal bleeding (HR = 0.97, 95%CI: 0.73–1.29) between patients treated with DOACs compared to patients treated with warfarin.

## Discussion

The main findings of our meta-analysis can be summarized as follows: (1) DOACs were associated with lower risks of SSE, ischemic stroke, hemorrhagic stroke, and all-cause death compared with warfarin. (2) In safety outcomes, DOACs therapy was associated with a significant reduction in major bleeding and intracranial hemorrhage but with no significant difference in the risk of gastrointestinal bleeding compared with warfarin.

Patients with AF and frailty have reduced physiological reserve and stress capacity, resulting in a substantially increased risk of thrombotic events, bleeding, and death than non-frail AF patients, making their management challenging. Frailty status was positively correlated with CHA2DS2-VASc and HAS-BLED scores, suggesting that frail patients may more urgently need OAC treatment to prevent stroke. Still, anticoagulant use often comes at the expense of a potential risk of bleeding (24). Balancing the benefits and risks of anticoagulation in such patients is a significant challenge for clinicians. Available studies confirmed that most frail patients, whether formally assessed or not, should receive OAC because the benefit outweighs the absolute risk of bleeding (25). However, guidelines do not give clear recommendations on the dosage and specific types of OAC to prescribe, and there is a paucity of relevant studies on frail patients.

Current AF guidelines recommend the use of DOACs as first-line drugs for stroke prevention based on four published remark DOAC trials. This means that the advantages of DOACs over warfarin in the general population have been well-proved. However, the anticoagulation strategies for stroke prevention in AF patients with co-morbidities (e.g., frailty, anemia, cancer) are incomplete, and further studies should confirm the advantages of DOACs in these special populations. In our meta-analysis, we synthesize evidence on treatment strategies in frail AF patients and provide some insights into the advantages of DOACs over warfarin. Due to the poor representation of frail patients and the lack of assessment of frailty, there were no relevant RCTs, so our meta-analysis selected relatively high-quality retrospective studies that covered a larger number of frail AF populations. There was considerable heterogeneity in the pooled estimates, and the heterogeneity of outcomes was not reduced by excluding one study at a time, indicating that the results were stable. Many reasons contribute to differences among studies, such as different thresholds for defining frail status, insufficient follow-up time, possible misclassification and selection bias in claims database-based studies, and exclusion of severely frail patients.

Perera et al. have shown that in geriatric medicine, general medicine, and cardiology services, frail AF patients were significantly less likely to use warfarin upon hospital admission and discharge than non-frail patients and appeared more vulnerable to adverse clinical outcomes, with and without antithrombotic therapy (8). In the meantime, high rates of morbidity and polypharmacy and the risk of falls are often common reasons for not using oral anticoagulants (OACs) in these patients (26). However, our study shows that DOACs are more effective and safer than conventional VKA-warfarin. This evidence will provide clinicians with firm support for anticoagulation in patients with AF and frailty, making it a promising candidate for the first choice of antithrombotic drugs in this population. We know that DOACs are directed against a single active coagulation factor. Its anticoagulant effect is independent of antithrombin, its pharmacokinetics are stable, and there are few interactions with food and drugs (27). This feature may make it more suitable for patients with AF and frailty who have deteriorated multiple physiological systems and require multiple medications. Because frail older patients are prone to decrease renal function, dabigatran has the highest renal clearance, which may lead to higher plasma concentrations of the drug, thereby increasing the risk of bleeding. In contrast, apixaban, which has lower renal clearance, appears to be safer. However, due to the absence of head-to-head clinical trials between DOCAs, our article cannot prove which DOACs are more effective and safer. Future research will help to provide robust evidence for this issue.

An interesting finding is that the studies we included reported the effects of different doses of DOACs. Research by Lip et al. showed that there was no statistically significant difference in the incidence of the primary effectiveness and safety outcome between patients taking standard-dose DOACs and the reduced-dose compared with warfarin (21). In contrast, Okumura et al. demonstrated in a randomized clinical trial that, in old Japanese patients (≥80 years of age) with NVAF, a once-daily 15-mg dose of edoxaban significantly reduced the risk of SSE and did not result in a significantly higher incidence of major bleeding (28). Subjects in this trial have the poor renal function, low body weight, a history of severe bleeding, ongoing use of non-steroidal anti-inflammatory drugs (NSAIDs), or current use of antiplatelet drugs, all of which present a dilemma for oral anticoagulation in these patients (29). However, edoxaban 15-mg daily provided them with strong oral anticoagulation support. It is reassuring that more and more research is beginning to focus on oral anticoagulation in old frail patients, further research will help to provide robust evidence for this issue.

## Limitation

Our study had several limitations. First, we included a limited number of observational studies, reducing the reliability of our findings. Second, the thresholds for defining frail status differed in each study, and subjects with different baseline characteristics may have significant bias despite statistical adjustments. At the same time, since most of the studies were based on claims databases, misclassification and selection bias may be responsible for the high heterogeneity of outcomes. Third, the different definitions of frailty cannot perform a detailed comparative analysis. Due to the small number of included studies, we were also unable to obtain sufficient data to perform a subgroup analysis of the results with high heterogeneity.

## Conclusion

In conclusion, for patients with AF and frailty, DOACs exerted superior effectiveness and safety outcome than warfarin in reducing the risk of SSE, ischemic stroke, hemorrhagic stroke, all-cause death, major bleeding, and intracranial hemorrhage. Still, there is no difference in gastrointestinal bleeding.

## Data Availability Statement

The original contributions presented in the study are included in the article/Supplementary Material, further inquiries can be directed to the corresponding author/s.

## Author Contributions

All authors listed have made a substantial, direct, and intellectual contribution to the work and approved it for publication.

## Funding

This study was funded by National Natural Science Foundation of China (21866019).

## Conflict of Interest

The authors declare that the research was conducted in the absence of any commercial or financial relationships that could be construed as a potential conflict of interest.

## Publisher's Note

All claims expressed in this article are solely those of the authors and do not necessarily represent those of their affiliated organizations, or those of the publisher, the editors and the reviewers. Any product that may be evaluated in this article, or claim that may be made by its manufacturer, is not guaranteed or endorsed by the publisher.
