# Supplementary material for: Effectiveness and Safety of DOACs vs. Warfarin in Patients With Atrial Fibrillation and Frailty: A Systematic Review and Meta-Analysis
Source: Front Cardiovasc Med. 2022 Jun 24;9:907197. doi: 10.3389/fcvm.2022.907197 (PMC9263568; doi:10.3389/fcvm.2022.907197)
Supplement: Supplementary file 1 [file Table_1.docx]

**Supplementary Table 1. The search strategies until March 2022**

|  | **Search terms** | **Pubmed** | **Embase** |
| --- | --- | --- | --- |
| #1 | atrial fibrillation | 96,605 | 200,585 |
| #2 | dabigatran | 6,201 | 19,240 |
| #3 | rivaroxaban | 7,219 | 23,582 |
| #4 | apixaban | 4,743 | 17,108 |
| #5 | edoxaban | 1,940 | 6,637 |
| #6 | Non-vitamin K oral anticoagulants | 1,853 | 622 |
| #7 | Direct oral anticoagulants | 7,897 | 7,063 |
| #8 | Novel oral anticoagulants | 2,771 | 2,113 |
| #9 | DOAC | 2,222 | 4,524 |
| #10 | NOAC | 1,728 | 3,805 |
| #11 | #2 OR #3 OR #4 OR #5 OR #6 OR #7 OR #8 OR #9 OR #10 | 19,057 | 41,793 |
| #12 | frail | 34,817 | 29,653 |
| #13 | frailty | 21,520 | 34,139 |
| #14 | frailness | 34,817 | 33 |
| #15 | Frailty Syndrome | 21,369 | 931 |
| #16 | #12 OR #13 OR #14 OR #15 | 34,956 | 51,227 |
| #17 | Vitamin K antagonists | 9,727 | 8,158 |
| #18 | VKA | 2,289 | 4,958 |
| #19 | warfarin | 32,492 | 104,946 |
| #20 | dicoumarol | 2,805 | 4,173 |
| #21 | acenocoumarol | 1,765 | 6,989 |
| #22 | coumadin | 32,968 | 5,032 |
| #23 | #17 OR #18 OR #19 OR #20 OR #21 OR #22 | 43,763 | 119,343 |
| #24 | #1 AND #11 AND #16 AND #23 | 74 | 184 |

**Supplementary Table 2. Quality assessment for the included post-hoc analyses of observational studies**

| Included studies | Selection (0-4 points) | | | | Comparability (0-2 points) | | Outcome (0-3 points) | | | Total poitns* |
| --- | --- | --- | --- | --- | --- | --- | --- | --- | --- | --- |
|  | Representativeness of Exposed Cohort | Selection of Non-Exposed Cohort | Ascertainment of Exposure | Demonstration That Outcome of Interest Was Not Present at Start of Study | Adjust for the important Risk factors | Adjust for other risk factors | Assessment of outcome | Follow-up length | Loss to follow-up rate |  |
| Martinez -2018 | * | * | * |  | * | * | * | * | * | 8 |
| Kim -2021 | * | * | * |  | * | * | * |  | * | 7 |
| Lip -2020 | * | * | * |  | * | * | * | * | * | 8 |
| Wilkinson-2020 | * | * | * |  | * | * | * | * | * | 8 |

＊The Newcastle-Ottawa Scale (NOS) items, with a total score of 9 points, were used to evaluate the quality of the post-hoc analyses of RCTs and observational study which involve the selection of cohorts (0-4 points), the comparability of cohorts (0-2 points), and the assessment of the outcome (0-3 point
